# Supplementary figures and images for: Structural model of microtubule dynamics inhibition by kinesin-4 from the crystal structure of KLP-12 –tubulin complex
Source: eLife. 2022 Sep 6;11:e77877. doi: 10.7554/eLife.77877 (PMC9451533; doi:10.7554/eLife.77877)

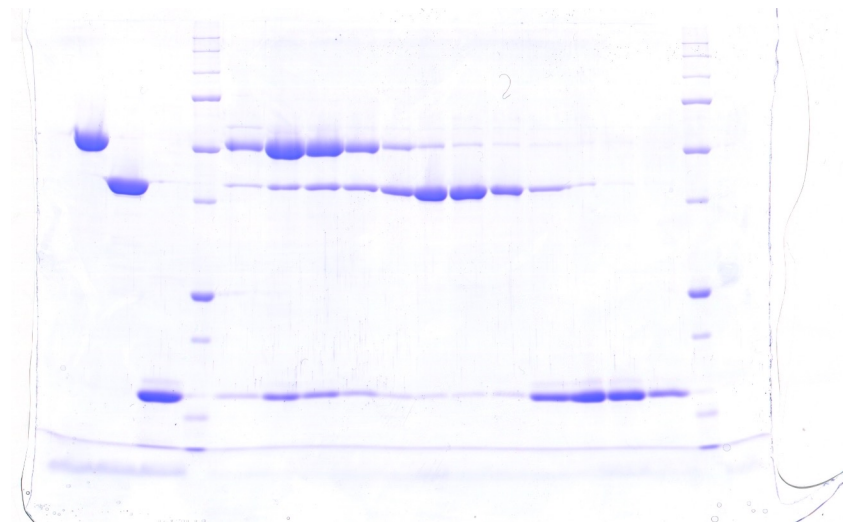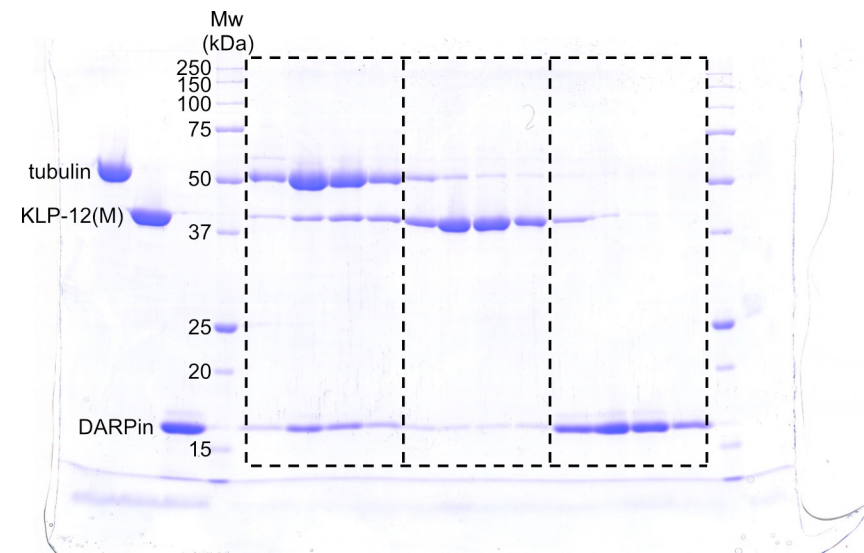

Supplement: Figure 3—source data 5. [file elife-77877-fig3-data5.pdf]

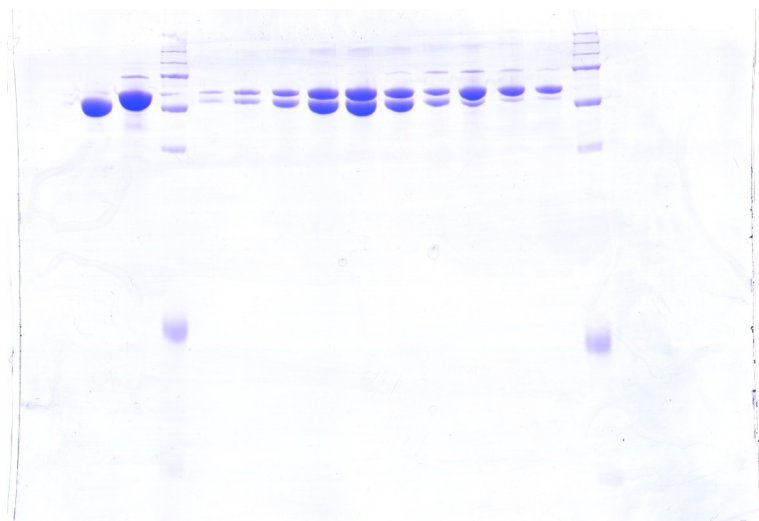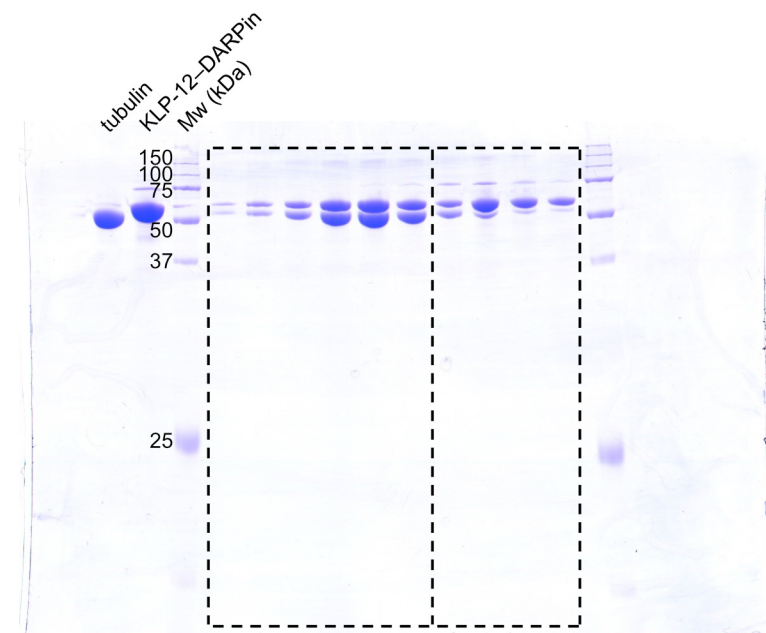

Supplement: Figure 3—figure supplement 2—source data 1. [file elife-77877-fig3-figsupp2-data1.pdf]
